# The intracellular domain of β-dystroglycan mediates the nucleolar stress response by suppressing UBF transcriptional activity

**DOI:** 10.1038/s41419-019-1454-z

**Published:** 2019-02-27

**Authors:** Paulina Margarita Azuara-Medina, Ariana María Sandoval-Duarte, Sara L. Morales-Lázaro, Ricardo Modragón-González, Griselda Vélez-Aguilera, Juan de Dios Gómez-López, Guadalupe Elizabeth Jiménez-Gutiérrez, Reynaldo Tiburcio-Félix, Ivette Martínez-Vieyra, Rocío Suárez-Sánchez, Gernot Längst, Jonathan Javier Magaña, Steve J. Winder, Arturo Ortega, Rita de Cassia Ramos Perlingeiro, Laura A. Jacobs, Bulmaro Cisneros

**Affiliations:** 1 Departamento de Genética y Biología Molecular, Centro de Investigación y de Estudios Avanzados Del Instituto Politécnico Nacional, 07360 Ciudad de México, Mexico; 2 Departamento de Neurociencia Cognitiva, Instituto de Fisiología Celular, Universidad Nacional Autónoma de México, 04510 Ciudad de México, Mexico; 3 Laboratorio de Hematobiología, Escuela Nacional de Medicina y Homeopatía, Instituto Politécnico Nacional, 07320 Ciudad de México, Mexico; 4 Laboratorio de Medicina Genómica, Instituto Nacional de Rehabilitación, 14389 Ciudad de México, Mexico; 5 Biochemistry Centre Regensburg (BCR), Universität Regensburg, 93053 Regensburg, Germany; 6 Department of Biomedical Science, University of Sheffield, Sheffield, S10 2TN UK; 7 Departamento de Toxicología, Centro de Investigación y de Estudios Avanzados Del Instituto Politécnico Nacional, 07000 Ciudad de México, Mexico; 8 Department of Medicine, Lillehei Heart Institute, University of Minnesota, Minneapolis, MN USA

## Abstract

β-dystroglycan (β-DG) is a key component of multiprotein complexes in the plasma membrane and nuclear envelope. In addition, β-DG undergoes two successive proteolytic cleavages that result in the liberation of its intracellular domain (ICD) into the cytosol and nucleus. However, stimuli-inducing ICD cleavage and the physiological relevance of this proteolytic fragment are largely unknown. In this study we show for the first time that β-DG ICD is targeted to the nucleolus where it interacts with the nuclear proteins B23 and UBF (central factor of Pol I-mediated rRNA gene transcription) and binds to rDNA promoter regions. Interestingly DG silencing results in reduced B23 and UBF levels and aberrant nucleolar morphology. Furthermore, β-DG ICD cleavage is induced by different nucleolar stressors, including oxidative stress, acidosis, and UV irradiation, which implies its participation in the response to nucleolar stress. Consistent with this idea, overexpression of β-DG elicited mislocalization and decreased levels of UBF and suppression of rRNA expression, which in turn provoked altered ribosome profiling and decreased cell growth. Collectively our data reveal that β-DG ICD acts as negative regulator of rDNA transcription by impeding the transcriptional activity of UBF, as a part of the protective mechanism activated in response to nucleolar stress.

## Introduction

Regulated proteolysis of cell surface receptors that liberates biologically active proteins/peptides from the plasma membrane (PM) to the cytosol is a critical step in a variety of different signaling pathways that respond to external stimuli. γ-Secretase is an intramembranous cleaving protease complex consisting of at least four proteins: presenilin-1, nicastrin, anterior pharynx-defective phenotype 1, and presenilin enhancer 2^1^. γ-Secretase is known to be required for the activation of many transmembrane proteins, including the amyloid precursor protein, cadherins, Notch1^2^, and recently, dystroglycan^3,4^. Dystroglycan, a key component of the dystrophin-associated protein complex (DAPC), is transcribed from the *DAG1* gene and translated as a single propeptide, which is proteolytically processed to generate the extracellular subunit α-dystroglycan (α-DG) and the transmembrane subunit β-dystroglycan (β-DG)^5^. α-DG binds to different extracellular matrix proteins including laminin, agrin, or perlecan^6^, while β-DG connects actin through various cytolinker proteins including dystrophin or utrophin. Thereby, dystroglycan serves as a link between the extracellular matrix and the actin-based cytoskeleton, acting also as an adhesion and signaling receptor^5,7^.

Besides its structural role in the maintenance of membrane integrity, dystroglycan localization is not static but dynamic. Phosphorylation of β-DG at Y^890^ triggers its retrograde trafficking from PM to the nucleus, via the membranous endosome-endoplasmic reticulum (ER) network, with ezrin activation enhancing the intracellular trafficking and translocon Sec61 facilitating the exit of β-DG from the ER membrane to be accessible for importin-dependent nuclear import through the nuclear pore^8–10^. In the nucleus, β-DG is assembled with nuclear envelope (NE) components, including emerin, and lamins A/C and B1, to preserve the nuclear structure/function^11,12^ and where it can also indirectly regulate gene expression^13^. This functional diversity of β-DG, acting as a platform for both PM- and NE-associated processes, is further expanded by proteolytic cleavage of the protein. β-DG is subjected to proteolytic cleavage by MMP-2 and MMP-9 to liberate its extracellular domain^14,15^, while the remaining fragment, containing the transmembrane stub and the cytoplasmic portion is thought to be subsequently processed by γ-secretase to deliver an intracellular domain (ICD; 12 kDa in mass but runs aberrantly on SDS-PAGE at ~26 kDa) into the cytosol^3,4^. Recent evidence showed that β-DG ICD is targeted to the nucleus in prostate cancer cells^3,13,16^ nonetheless the biological significance of such localization is largely unknown. The nucleus is organized into distinct functional compartments containing specific macromolecules that govern nuclear processes;^16^ for instance, the nucleolus is a prominent non-membranous nuclear organelle primarily involved in ribosome biogenesis and cellular homeostasis^17^. Thus, identification of the destination of β-DG ICD within the nucleus could facilitate further elucidation of its function.

In this study we demonstrate for the first time that β-DG ICD is target to the nucleolus where it plays a negative role in the regulation of ribosomal RNA (rRNA) transcription. We provide evidence that full-length β-DG is proteolytically processed into β-DG ICD in response to nucleolar stress, via the Notch signaling pathway. Remarkably, β-DG ICD binds to the rDNA promoter to suppress rRNA synthesis by impairing the expression, localization, and ultimately activity of the RNA polymerase I (Pol I) transcription factor UBF (upstream binding factor), which further results in the downregulation of rRNA expression and cell proliferation. Thus, β-DG ICD appears to be a key contributor to the nucleolar stress response.

## Results

### The γ-secretase-generated intracellular domain of β-DG is targeted to the nucleolus

**Fig. 1:**
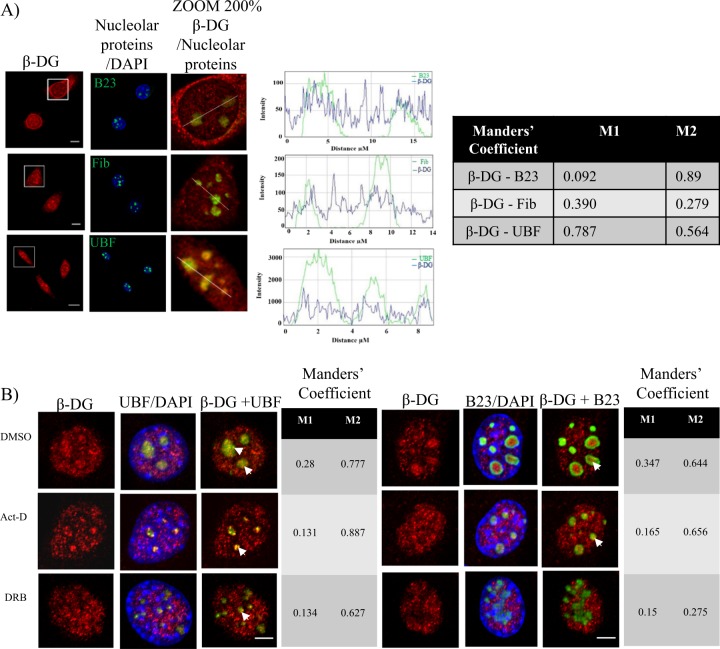
β-DG colocalizes with nucleolar proteins before and after induced nucleoli disorganization. **a** C2C12 cells were double immunostained for β-DG (C20 antibodies) and B23, fibrillarin (Fib), or UBF. Cells were stained with DAPI for nuclei visualization prior to being subjected to confocal laser scanning microscopy (CLSM; scale bar = 5 µm). Colocalization of β-DG with nucleolar proteins is denoted by arrowheads in the magnified images. A line intensity scan along the white line in the merge images was carried using ROI manager Multiplot (middle graphs). *Right*: The Manders overlap coefficient was calculated on double labeling inmunofluorescences: M1 denotes the fraction of the β-DG signal coincident with the nucleolar proteins signal over its total intensity, and M2 denotes the fraction of the nucleolar proteins signal coincident with the β-DG signal over its total intensity. **b** Cells were treated with actinomycin D (Act D) or DRB for 3 h, fixed and then double immunostained for β-DG and either UBF or B23. Nuclei were stained with DAPI before CLSM analysis (scale bar = 1 µm). Typical single Z-sections are shown and colocalization of β-DG with nucleolar proteins after drug-induced nucleoli disruption is indicated by arrowheads in the merge images. The Manders overlap coefficient was calculated for each experimental condition as peer **a**

We previously observed localization of β-DG to the nucleoli in C2C12 myoblasts^11^; but no role for β-DG has been described in this nuclear organelle. As a first step, we analyzed whether β-DG colocalizes with proteins that define functionally distinct compartments of the nucleolus. Cells were double-stained for β-DG (C20 antibody) along with UBF, fibrillarin (markers of the fibrillar center, FC), or B23 (marker of the granular component, GC) and further analyzed by confocal microscopy. The nucleolar immunostaining of β-DG colocalized at certain extent with all three nucleolar proteins analyzed, as confirmed by the line intensity scan analysis and Manders’ overlapping coefficients (Fig. 1a). The specificity of C20 antibody was demonstrated using both DG knockout C2C12 cells and primary fibroblasts from a subject with Walker–Warburg syndrome that do not express *DAG1* gene all^18^. Nucleoli were strongly staining by C20 in C2C12 wild-type cells, while a faint nuclear staining that was excluded from nucleoli was observed in DG knockout C2C12 cells (Supplementary Figure 1A, left panel). Furthermore, the immunoreactive band corresponding to β-DG (~43 kDa), which was observed in primary fibroblast lysates from a healthy subject, was absent in Walker–Warburg syndrome primary fibroblast lysates (Supplementary Figure 1A, right panel). Finally, pre-incubation with a blocking peptide virtually eliminated C20 immunostaining in wild-type cells (Supplementary Figure 1B). In addition, nucleolar localization of β-DG was confirmed using another anti-β-DG antibody (G5; Supplementary Figure 1C). The nucleolus is a highly dynamic organelle that responds to a variety of cellular stresses with morphological changes^19^ and redistribution of proteins implicated in ribosome biogenesis form perinuclear caps upon inhibition of rDNA transcription^20^. C2C12 cells were therefore treated with actinomycin D (0.08 µg/ml) or DRB (0.3 µM) to cause nucleolar stress, and the effect of treatments on β-DG distribution was evaluated by confocal microscopy. The formation of nucleolar caps and nucleolar segregation/nucleolar necklaces was found after treatment with Act D and DRB, respectively, as shown by immunostaining for UBF and B23 (Fig. 1b). Treatment with the vehicle alone dimethyl sulfoxide (DMSO) produced no change in the immunostaining pattern of nucleolar proteins. Interestingly, nucleolar labeling of β-DG also underwent redistribution in response to Act D and DRB but remained in colocalization partially with UBF and barely with B23, as shown by Manders’ overlapping coefficient analysis (Fig. 1b), suggesting that β-DG might be involved in nucleolar organization/plasticity. Next, we investigated whether the nucleolar localization of β-DG is dependent on nucleic acid integrity. C2C12 myoblasts were immunolabeled for β-DG before and after nuclease treatment, and stained with DAPI to monitor DNA degradation, or immunolabeled for hnRNP C1/C2 antibody (RNA-binding protein) to monitor RNA removal. DNase digestion completely removed DAPI-labeled DNA, while treatment with RNase caused hnRNP C1/C2 redistribution from the nucleoplasm to the nuclear periphery and cytoplasm, thus confirming the effectiveness of nucleases treatment. DNase treatment completely abrogated the nucleolar distribution of β-DG, while treatment with RNase apparently increased the intensity of β-DG nucleolar immunostaining, compared with untreated cells (Supplementary Figure 2A and B). These results suggest that the nucleolar localization of β-DG is dependent on DNA.

**Fig. 2:**
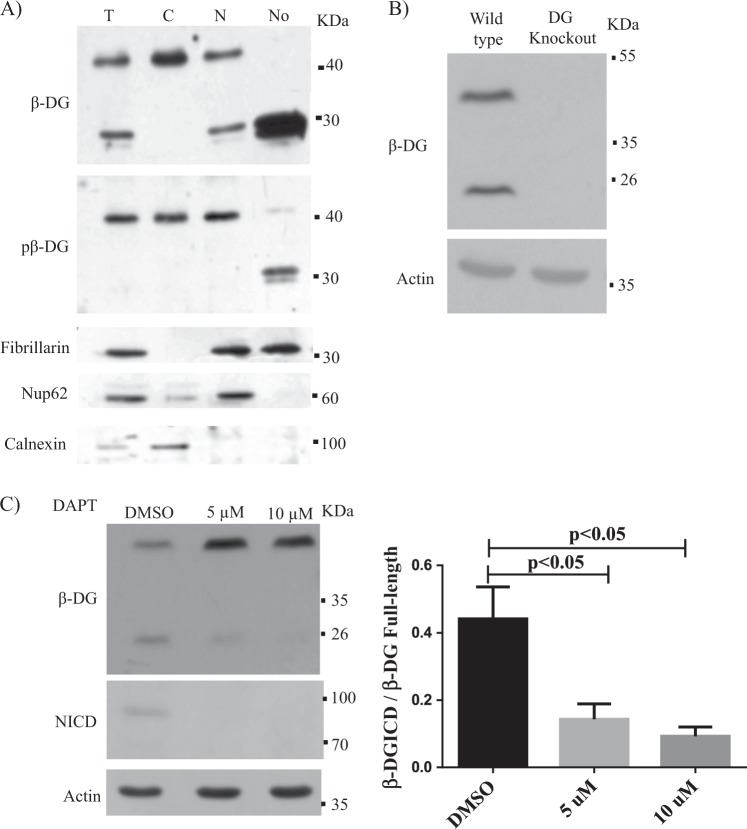
β-DG ICD resides in the nucleolus. **a** Cells were partitioned to obtain total (T), cytoplasmic (C), nuclear (N), and nucleolar (No) fractions, prior to SDS-PAGE/WB analysis, using specific antibodies for β-DG (Mandag) or its phosphorylated counterpart (pβ-DG). Fibrillarin (nucleolar protein), Nup62 (nuclear membrane protein), and calnexin (reticulum endoplasmic protein) were used as cellular fraction markers. **b** Lysates from wild-type and DG knockout C2C12 cells were analyzed by SDS-PAGE/WB using antibodies for β-DG (Mandag) and actin (loading control), to demonstrate β-DG antibody specificity. **c** Lysates from C2C12 cells treated for 24 h with the indicated concentrations DAPT (γ-secretase inhibitor) or the vehicle alone (DMSO) were analyzed by SDS-PAGE/WB, using antibodies for β-DG, Notch intracellular domain (NICD; positive control for DAPT action), and actin (loading control). Representative gel from three independent experiments is shown. *Right*. Data correspond to the mean ± SEM from three independent experiment, with *p* values indicating significant differences (unpaired *t*-test)

To demonstrate conclusively that β-DG resides in the nucleolus, C2C12 were fractionated into total, cytoplasmic, nuclear, and nucleolar fractions to be further analyzed by SDS-PAGE/WB. A prominent ~30 kDa β-DG band was found enriched, together with fibrillarin, in the nucleolar fraction, as revealed using three different anti-β-DG antibodies: MANDAG (mouse mab that recognizes total β-DG levels), pTyr890 (rabbit pAb that specifically recognizes β-DG phosphorylated at Tyr^890^) (Fig. 2a), and C20 (rabbit antibody that recognizes the total β-DG pool Supplementary Figure 1D). Nup62 (nuclear pore protein) and calnexin (endoplasmic reticulum protein) were recovered in the nuclear and cytoplasmic fractions, respectively, and the two proteins were absent from the nucleolar fraction demonstrating that no cross-contamination occurred during cellular fractionation. Control experiments using both DG knockout C2C12 cells and Walker–Warburg syndrome primary fibroblasts yield no immunoreactive bands after incubation with MANDAG antibody (Fig. 2b and Supplementary Figure 1A, right panel), which demonstrated the specificity of this antibody for β-DG detection. We hypothesized that the ~30 kDa band correspond to the ICD of β-DG generated by γ-secretase cleavage as has been described previously^3,13,16^. Compatible with this assumption, lysates from cells treated with DAPT (γ-secretase inhibitor) resulted in a dose-dependent decrease in β-DG ICD levels that paralleled with dose-dependent reduction of the intracellular domain of Notch (NICD), a well-characterized γ-secretase substrate (Fig. 2c). Collectively these data support the idea that the γ-secretase-derived cleavage fragment of β-DG (~30 kDa), named hereafter as β-DG ICD, is targeted to the nucleolus where it possibly has a role in regulating nucleoli structure/function.

### β-DG ICD interacts with UBF and B23

**Fig. 3:**
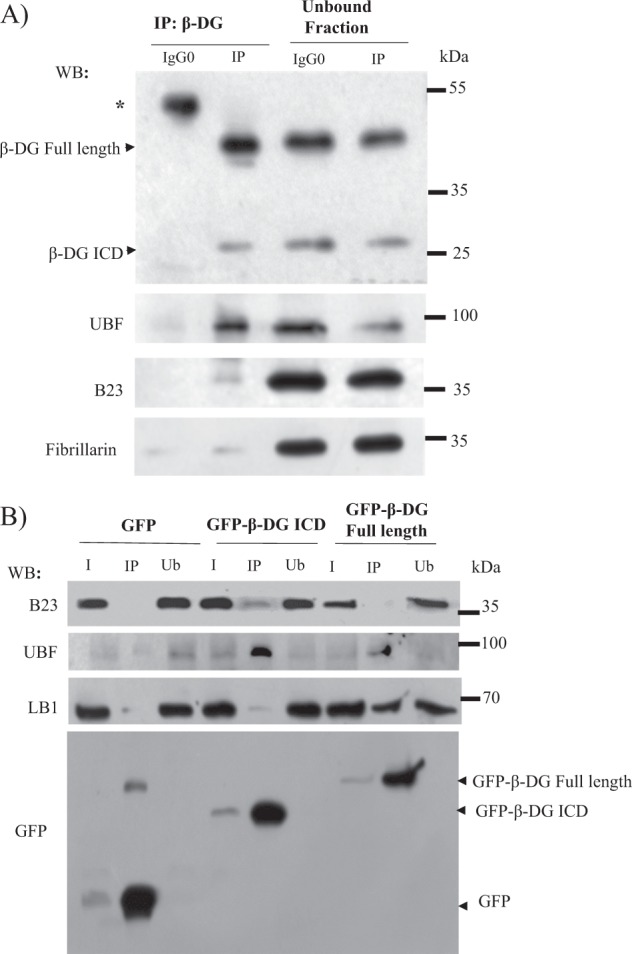
β-DG ICD interacts with the nucleolar proteins UBF and B23. **a** C2C12 cell lysates were subjected to immunoprecipitation (IP) using anti-β-DG antibody (Mandag) and the precipitated proteins were subsequently analyzed by western blotting using primary antibodies against β-DG, UBF, B23, or fibrillarin. **b** Lysates from cells transiently expressing GFP-β-DG full-length, β-DG ICD, or GFP alone were subjected to immunoprecipitation using the GFP-Trap system, and the captured proteins were analyzed by western blotting using antibodies against B23, UBF, lamin B1 (LB1), and GFP. **a**, **b** Ub, unbound proteins, IP immunoprecipitated fraction, IgG0, nonspecific antibody. Input corresponds to 10% of protein lysates prior to immunoprecipitation

Colocalization of β-DG with nucleolar proteins even after induced nucleolar disruption (Fig. 1b), suggests their physical interaction. Consistent with this notion, UBF and traces of B23 but not fibrillarin were recovered in complex with β-DG after immunoprecipitation (IP) of C2C12 lysates with anti-β-DG antibody (Fig. 3a). None of the nucleolar proteins were immunoprecipitated using nonspecific IgG antibodies. Because anti-β-DG antibody immunoprecipitated both β-DG full-length and β-DG ICD, we wondered what form is bound to nucleolar proteins. To approach this, C2C12 cells were transiently transfected to express GFP-β-DG full-length or GFP-β-DG ICD and subjected to GFP-based IP assays. Both UBF and B23 were found preferentially bound to GFP-β-DG ICD, with UBF interaction predominant over that with B23 (Fig. 3b). GFP alone interacted with neither B23 nor UBF, while lamin B1 (β-DG nuclear partner) was bound to GFP-β-DG full-length but not GFP-β-DG ICD, demonstrating the specificity of GFP-Trap assays.

### Silencing of DG disrupts nucleolar morphology and decreases B23 and UBF levels

**Fig. 4:**
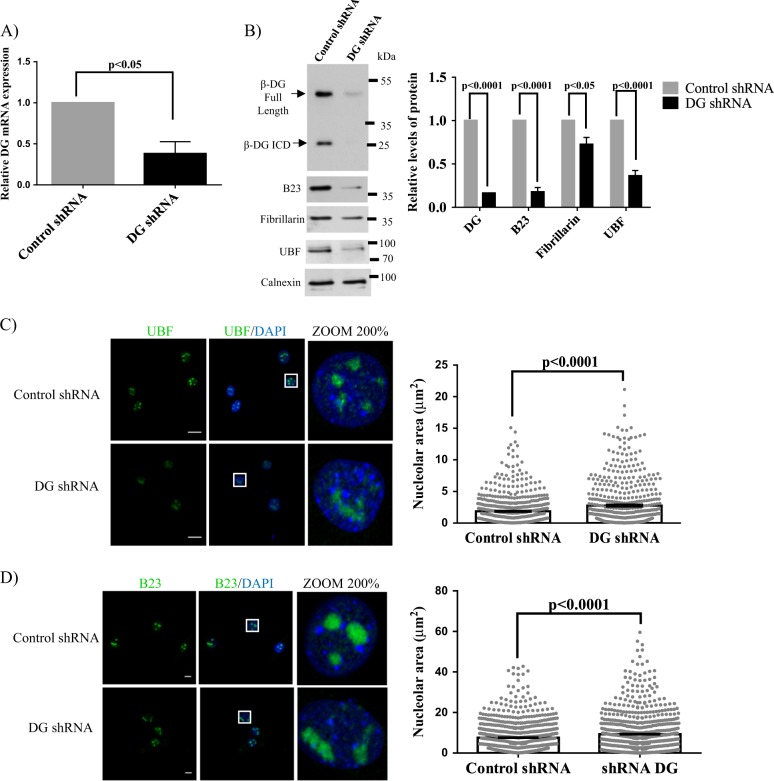
Silencing of dystroglycan results in altered nucleoli structure and reduced levels of B23 and UBF. **a** Messenger RNA expression of dystroglycan and GAPDH (endogenous control) was assessed by qRT-PCR in C2C12 cells stably expressing DG shRNA or control shRNA, as described in Methods. Relative mRNA levels obtained in control cells were set at 1. Results are the mean ± standard deviation of three independent experiments, with *P* value indicating significance difference (unpaired *t*-test). **b** Lysates from C2C12 cells stably transfected with DG shRNA or control shRNA were analyzed by western blotting using primary antibodies against DG, B23, fibrillarin, UBF, and calnexin (loading control). Blots were stripped and reprobed successively with each protein antibody. Relative protein levels from control shRNA cells were set at 1 for comparison. Results are the mean ± SEM of three independent experiments (right panel), with *p* value denoting significance differences (unpaired *t*-test). **c**, **d** Nucleolar morphology was analyzed in cells stably expressing DG shRNA or control shRNA, using specific antibodies against UBF and B23 respectively. Cells were counterstained with DAPI to visualize nuclei prior to CLSM analysis. The nucleolar area (μm^2^) was determined in each cell condition as described in Methods (graphs at right). Data are the mean ± SEM from three independent experiments (*n* = 600 nucleoli), with *p* values indicating significant differences (unpaired *t*-test)

To elucidate whether nucleolar localization of β-DG is physiologically relevant, the impact of knocking down of β-DG expression on nucleolar structure and expression of nucleolar proteins was examined. Significant reduction of dystroglycan mRNA levels and the consequent decrease in β-DG full-length and β-DG ICD levels were observed upon stably transfection of C2C12 cells with a vector expressing a short hairpin RNA against *DAG1* gene (DG shRNA), compared to cells expressing an unspecific shRNA (control shRNA) (Fig. 4a, b). Remarkably, the depletion of β-DG resulted in decreased proteins levels of B23 (80%), fibrillarin (30%), and UBF (60%) (Fig. 4b). Such effects were accompanied by a clear alteration in nucleolar morphology; while control shRNA cells contain numerous relatively small nucleoli, β-DG-depleted cells exhibited fewer larger and more amorphous nucleoli, as shown by immunostaining for UBF and B23 (Fig. 4c, d) and quantification of nucleolar area (right panels).

### β-DG binds to the rDNA promoter to possibly regulate rRNA expression

**Fig. 5:**
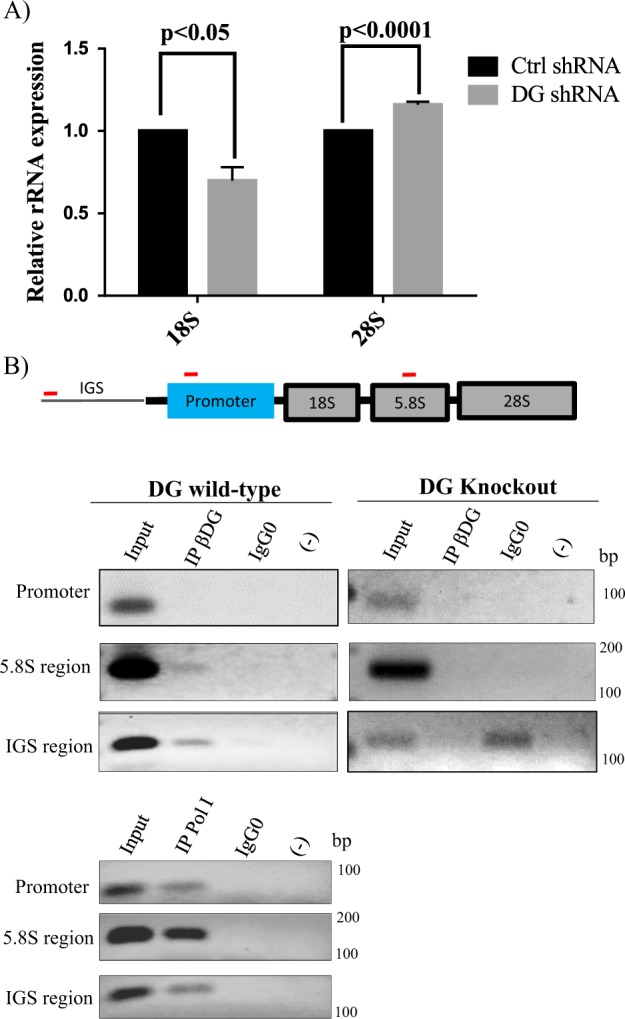
β-DG binds to the rDNA gene regulatory region and knockdown of DG expression alters rRNA expression. **a** Total RNA was isolated from control shRNA and DG shRNA stably transfected cells and the expression of 18 S and 28 S pre-rRNAs was assessed by qRT-PCR using GAPDH as endogenous control. The expression levels obtained in control shRNA cells were set at 1 for comparison. Data correspond to the mean ± SEM from three independent experiments with duplicates; *p* values denoted significant differences (unpaired *t*-test). **b** Wild-type and DG knockout C2C12 cells were subjected to chromatin immunoprecipitation (ChIP) with antibody anti-β-DG (MANDAG), followed by PCR assays for the promoter, 5.8 S and IGS (intergenic sequence) regions of rDNA gene. ChIP assays using anti-PolI antibody were carried out in wildtype C2C12 cells as positive control. The drawing at the top shows the organization of the rDNA gene (bottom panel). IgG0 nonspecific antibodies, (-) mock reaction without antibodies

Because knockdown of β-DG resulted in decreased levels of UBF, a key component of the RNA polymerase I preinitiation complex in rRNA transcription, we sought to determine the expression of 28 S and 18 S pre-rRNAs in β-DG knockdown cells by quantitative RT-PCR (Figure 4a). β-DG knockdown cells exhibited an ~40% decrease in 18 S levels, while 28 S expression showed a slight but significant increase, compared with control shRNA (Fig. 5a). To provide insight into the mechanism by which β-DG depletion influences rDNA gene expression, we analyzed whether β-DG is physically associated with the rDNA promoter. Chromatin was immunoprecipitated from wild-type and DG knockout (negative control) C2C12 cells, using β-DG or nonspecific IgG antibodies; moreover, ChIP assays using RNA polymerase I (Pol I) antibodies were performed in parallel (positive control). Then, rDNA was amplified using primers specific for the rDNA promoter, 5.8 S or intergenic spacer (IGS) regions. ChIP assays revealed the presence of β-DG in the 5.8 S and IGS regions, while Pol I was found to occupy all three regions of the rDNA (Fig. 5b). As expected, no PCR amplification was detected upon ChIP assays on DG knockout cells (right panel), which demonstrated the specificity of Mandag antibody for immunoprecipitating β-DG from chromatin. Overall these data suggest that β-DG may modulate rRNA transcript expression through its binding to the rDNA regulatory region.

### Nucleolar stress-induced β-DG ICD cleavage correlates with impaired ribosome biogenesis

**Fig. 6:**
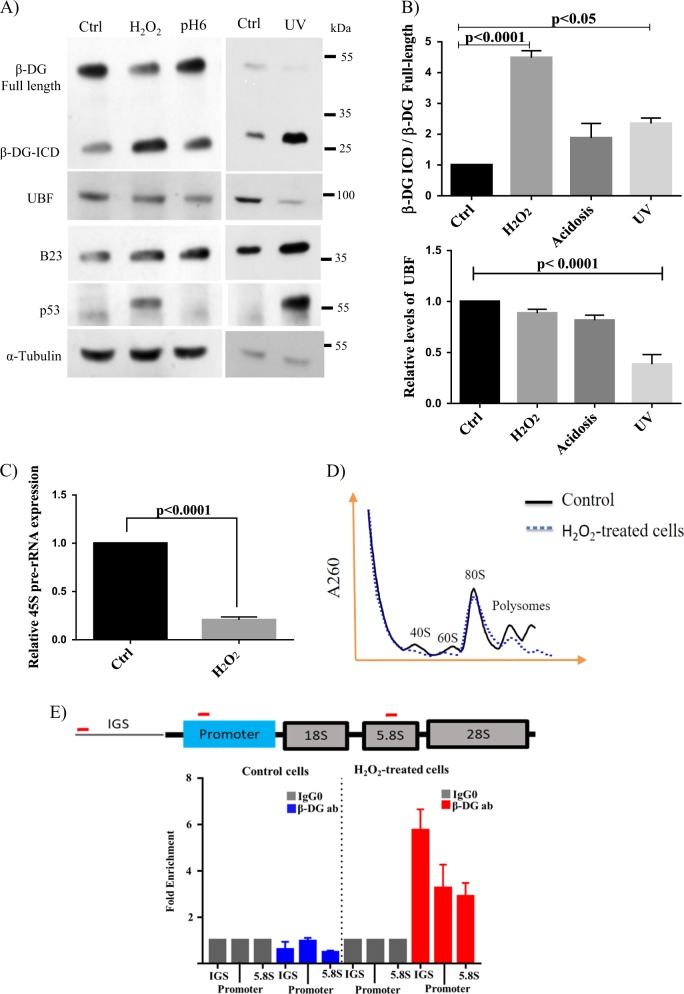
Nucleolar stress triggers β-DG ICD and disturbs rRNA expression and ribosome profile. **a** Lysates from control (ctrl, untreated) cells or cells induced to nucleolar stress response by H_2_O_2_ treatment, UV irradiation or acidosis (pH 6) (see Methods for details) were analyzed by western blotting using specific antibodies against β-DG, UBF, B23, p53, and α-tubulin. **b** Quantification of β-DG ICD/β-DG full-length ratio and UBF levels was carried out using α-tubulin as loading control. Results correspond to the mean ± SEM of three independent experiments with *p* values denoting significant differences (ordinary one-way ANOVA). **c** The 45 S rRNA precursor expression was assessed by qRT-PCR using GAPDH as endogenous control. The expression levels obtained in control cells were set at 1 for comparison. Data correspond to the mean ± SEM from three independent experiments with duplicates; *p* values denoted significant differences (unpaired *t*-test). **d** Ribosome profiling from control and H_2_O_2_-treated cells are shown. **e** Chromatin immunoprecipitation assays on control and H_2_O_2_-treated C2C12 cells were performed, with specific antibody against β-DG (Mandag), followed by SYBR green-based qPCR assays for the promoter, 5.8 S and IGS (intergenic sequence) regions of rDNA gene. Scheme of rDNA gene regulatory regions is shown at the top

In an effort to understand the potential role of β-DG ICD in the nucleolus, we investigated whether β-DG ICD cleavage is induced by nucleolar stress. C2C12 cells were subjected to different kind of stresses, namely oxidative stress, acidosis, and UV irradiation, and effectiveness of treatments was monitored by p53 activation. Interestingly, the ratio of β-DG ICD/full-length β-DG was augmented in response to all treatments with concomitant decrease in UBF content, with H_2_O_2_ and UV being the most efficient inductors (Fig. 6a, b). As expected, H_2_O_2_-induced nucleolar stress resulted in nucleolar segregations, with a formation of UBF-stained nucleolar caps at the periphery of B23-stained nucleoli (Supplementary Figure 3), which elicited ultimately downregulation of 45 S rRNA expression (Fig. 6c) and abnormal ribosomal profiling characterized by decreased monosomes and polysome absorbance peaks (Fig. 6d). We hypothesized that augmented β-DG ICD levels could be reflected in a higher binding activity to the rDNA promoter; consistent with this idea, occupancy of the three rDNA regulatory regions by β-DG markedly increased after H_2_O_2_ treatment, as revealed by ChIP-qPCR assays (Fig. 6e).

### Overexpression of β-DG ICD impairs ribosomal biogenesis by suppressing UBF function

**Fig. 7:**
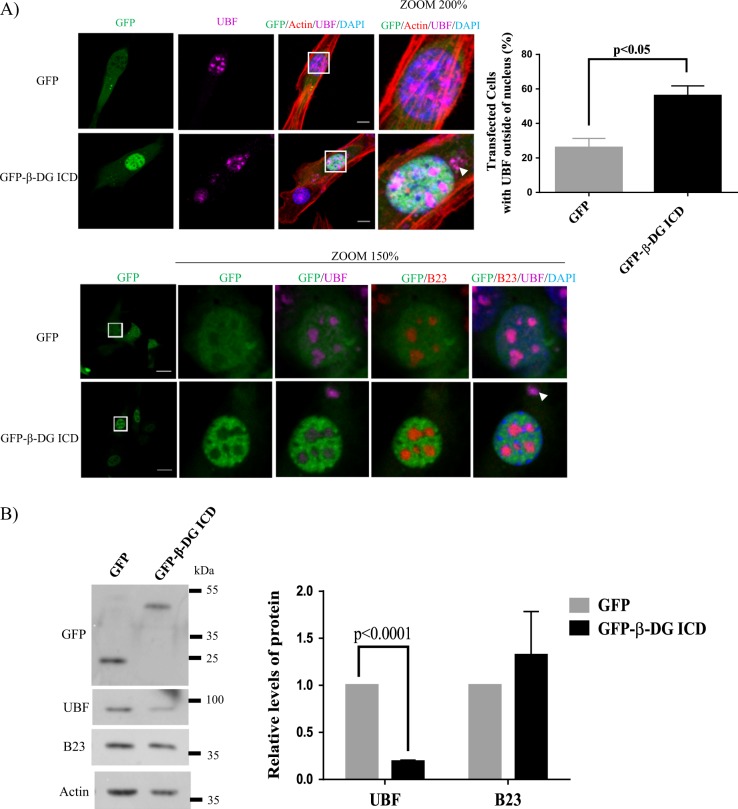
Overexpression of β-DG ICD induces mislocalization and reduces UBF levels. **a** C2C12 cells transiently expressing GFP or GFG-β-DG ICD were immunolabeling for UBF at 24 h post-transfection. Cells were then fixed, stained with phalloidin and DAPI to visualize actin and nuclei, respectively, and subjected to CLSM analysis. In parallel experiments transfected cells were double immunostained for UBF and B23 and counterstained with DAPI to visualize nuclei prior to CLSM analysis (bottom panels). Scale bar = 10 µm. Mislocalized UBF is denoted by arrowheads. Quantification of cells transfected with GFP and GFP-β-DG ICD that showed UBF outside of the nucleus (right panel). Results correspond to the mean ± SEM of three independent experiments with *p* values denoting significant differences (unpaired *t*-test), *n* = 50 per experiment. **b** Lysates from GFP- or GFG-β-DG-ICD-transfected cells were analyzed by western blotting using specific antibodies against GFP, UBF, B23, and actin (loading control). *Right*: Data correspond to the mean ± SEM from three independent experiments, with *p* values indicating significant differences (unpaired *t*-test)

**Fig. 8:**
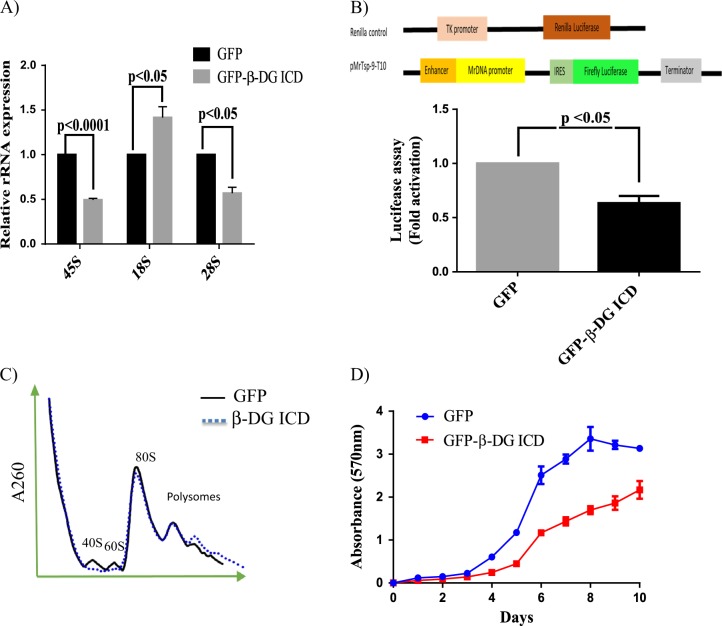
Exogenous expression of β-DG ICD impairs ribosome biogenesis by affecting transcriptional activity of UBF. C2C12 cells were transfected to stably express GFP-β-DG ICD or GFP alone (empty vector). **a** The expression of 45 S, 18 S, and 28 S rRNAs was analyzed in the transfected cell cultures, using GAPDH as endogenous control. **b** Schematic representation of the mouse rDNA promoter construct (pMrTsp-9-T10) and *Renilla* luciferase control vector is shown at the top. Cell cultures stably expressing GFP-β-DG ICD or GFP alone were co-transfected with both luciferase reporter constructs and transcriptional activities were estimated after incubation for 48 h, as described in Methods. Control condition obtained in cells transfected with GFP alone was set at 1 for comparison. Data represent mean ± SEM of three independent experiments; *p* values denote a significant difference (unpaired *t*-test). **c** The ribosomal gradient profiles obtained from transfected cell cultures are shown. **d** Cell proliferation of the stably transfected cell cultures was monitored over a 10-day period using the MTT assay. Data represent mean ± SEM of three independent experiments

To analyze directly the implication of β-DG ICD in ribosome biogenesis, we stably transfected C2C12 cells with a GFP-β-DG ICD vector, to mimic high levels of the cleavage fragment. GFP-β-DG ICD localization was restricted to the nucleus showing homogenous distribution throughout the nucleoplasm, with less intense staining in nucleoli (Fig. 7a). Remarkably, the significant number of GFP-β-DG ICD-transfected cells exhibited mislocalization of UBF foci to the cytoplasm, compared to GFP alone (50% and 20%, respectively), while the nucleolar distribution of B23 remained unaltered (Fig. 7a, bottom panel and right panel). Impaired nucleolar localization of UBF due to exogenous β-DG ICD expression was confirmed by transfecting FLAG-tagged β-DG ICD (Supplementary Figure 4, top rows). Furthermore, overexpression of GFP-β-DG ICD resulted in decreased levels of UBF with no effect on B23, compared to GFP alone (Fig. 7b and right panel). We have demonstrated previously that the phosphorylation of β-DG at Tyr890 is not only a signal for the proteolysis and internalization of dystroglycan^3,16,22^ but also acts to target dystroglycan to the nucleus^10^. We considered therefore whether phosphorylation of β-DG ICD at the PPxY motif (Tyr^890^) plays a role in UBF mislocalization. Thus, FLAG-β-DG ICD variants that either mimic (Y890E) or block (Y890A) the Tyr^890^ phosphorylation were analyzed for UBF localization. Similar percentage of transfected cells showing UBF outside of the nucleus were observed between β-DG ICD FLAGY890A and β-DG ICD-FLAGY890E; thus, overexpression of β -DG ICD resulted in UBF mislocalization irrespective of a charged residue at residue 890 that mimics phosphorylation (Supplementary Figure 4). We hypothesized that decreased expression/mislocalization of UBF through β-DG ICD overexpression would ultimately impact rRNA expression. Consistent with this idea, an ~50% decrease in 45 S rRNA and 28 S but not 18 S was found in cells stably overexpressing β-DG ICD, compared with those expressing GFP alone (Fig. 8a). To ascertain whether β-DG ICD overexpression influences Pol I transcription, functional rRNA promoter reporter assays were carried out in β-DG ICD stably transfected cells, using a vector that expresses Firefly luciferase under the control of the mouse rRNA promoter and a vector that expresses Renilla luciferase to normalize transfection efficiency. Pol I promoter activity was found to decrease by 40% in GFP-β-DG ICD-transfected, compared with those with GFP alone (Fig. 8b). Because the rRNA content is a crucial point of regulation for ribosome biogenesis, we hypothesized that decreased 40 S and 60 S levels would impact the ribosome profile. Consistent with this notion, altered ribosome profiles devoid of the peaks corresponding to free 40 S and 60 S ribosomal subunits were observed in cells expressing β-DG ICD (Fig. 8c). Such impairment in rRNA expression and polysome profiling ultimately leads to defective proliferative capacity of β-DG ICD overexpressing cells, as shown by MTT-based proliferation assays (Fig. 8d). Collectively these data indicate that β-DG ICD acts as negative regulator of rRNA expression by affecting both the levels and transcriptional activity of UBF.

## Discussion

β-DG is a key component of both PM and NE, acting as a platform for the proper anchorage of organelle-specific protein assemblies. At the PM β-DG modulates adhesion/signaling by connecting extracellular matrix proteins with the actin-based cytoskeleton^5,7^, while in the nucleus β-DG interacts with the NE proteins emerin and lamins A/C and B1 to regulate nuclear structure and function^11,12^. This functional diversity could be wider than originally thought, due to the potential function of β-DG proteolytic fragments. Recent in vitro and in vivo evidence showed that β-DG undergoes two successive and possibly coordinate proteolytic cleavages that result in the liberation of the intracellular domain (ICD) into the cytosol; first matrix metalloproteinases, MMP-2 and MMP-9 cleave the extracellular domain of β-DG^23^ and create a membrane-tethered intermediate that is subsequently processed by γ-secretase, a PM-embedded protease complex, to render a final cleavage product that approximates the entire ICD^21^. Except for cell density^21^, other cellular stimuli inducing β-DG ICD release as well as the physiological consequences of such processing event are largely unknown. In this study we showed that β-DG ICD associates to nucleoli and its cleavage is promoted by the induction of a different kind of nucleolar stresses. We also ascribed for the first time a role for β-DG ICD in regulating rRNA transcription through negative regulation of the expression and transcriptional activity of UBF, in the context of the response to nucleolar stress.

We show herein that β-DG colocalizes and interacts with the key nucleolar proteins B23 and UBF, and, based on the converging redistribution of β-DG with B23, fibrillarin, or UBF in response to both actinomycin- and DRB-induced nucleoli disorganization, we hypothesized that β-DG participates in the functional plasticity of nucleoli. Interestingly, cell fractionation experiments revealed the presence of β-DG ICD but not β-DG full-length in the nucleolar fraction of C2C12 cells; however, GFP-based IP experiments revealed that both β-DG ICD and β-DG full-length are able to bind UBF. Because full-length β-DG is an NE protein associated with nuclear lamin and because the interaction of intranuclear filaments of lamins with nuclear compartments (including nucleoli) has been well established^24–26^ it is possibly that IP of β-DG full-length pulled down a nuclear macromolecular complex containing NE (lamin B1) and nucleolar (UBF) proteins. The trafficking pathway underlying nucleolar targeting of β-DG ICD remains to be deciphered. Full-length β-DG is translocated to the nucleus through recognition of its NLS by importins α2/β1^9^, therefore it is likely that β-DG ICD enters the nucleus by virtue of the NLS that is still present in the cleaved fragment, or alternatively by passive diffusion through the nuclear pore complex (NPC), because its molecular mass (<30 kDa) is clearly below the NPC permissive size^27^. No obvious nucleolar localization signal is found in β-DG ICD, thus, its nucleolar accumulation might be mediated by a retention mechanism via interaction with nucleolar components. Supporting this idea, β-DG ICD was found to interact with B23, a shuttle protein that transports cargos between the cytoplasm and the nucleolus and mediates nucleolar retention of other proteins^28^. Furthermore, binding of β-DG to the rDNA gene regulatory region (see below) could serve to anchor β-DG ICD to nucleoli too. Owing to the main contribution of nucleoli in ribosome biogenesis^16^, we wondered whether β-DG is involved in this process. We provide here solid evidence supporting this hypothesis: first, β-DG interacts with UBF, an HMG box-containing protein that binds directly with rDNA and plays an important role in the recruitment of SL1 (selectivity factor 1) and Pol I to the rRNA promoter to constitute the Pol I preinitiation machinery complex^29^; second, ChIP assays with anti-β-DG antibody demonstrated β-DG recruitment to the rDNA 5.8 S and IGS regulatory regions; and third, DG silencing resulted in reduced UBF levels, decreased 18 S pre-rRNA expression but not 28 S pre-rRNA expression, and aberrant nucleolar morphology. Discordant expression between 28 S and 18 S rRNAs is intriguingly. Even though both 28 S rRNA and 18 S rRNA are originated from 45 S rRNA precursor, their processing/maturation pathways differ from each other, and each one is assisted by specific exo and endoribonucleasas^30^. Therefore, we argue that aside from its effect on rDNA basal transcription machinery (as shown here), β-DG ICD might modulate processing pathways of 18 S rRNA and 28 S rRNA differentially by indirect mechanisms. Additional experiments are needed to clear this issue.

The nucleolus is a central hub for orchestrating stress response, with downregulation of rRNA synthesis taking place as a part of ribosome biogenesis surveillance^31^, furthermore, numerous proteins that functions are not generally ascribed to ribosome biogenesis are recruited to the nucleolus in response to environmental stimuli^32^. Therefore, we ascertained whether β-DG ICD cleavage is coupled to the response to nucleolar stress. Remarkably, triggers of β-DG ICD cleavage by Notch signaling activation occurred in response to different nucleolar stressor treatments, including oxidative stress, acidosis, and UV irradiation. Supporting a role for β-DG ICD in the response to nucleolar stress, β-DG ICD overexpression was sufficient to elicit several features of nucleolar stress, including mislocalization and decreased levels of UBF, repression of rRNA transcription, altered ribosome profile, and decreased cell proliferation.

**Fig. 9:**
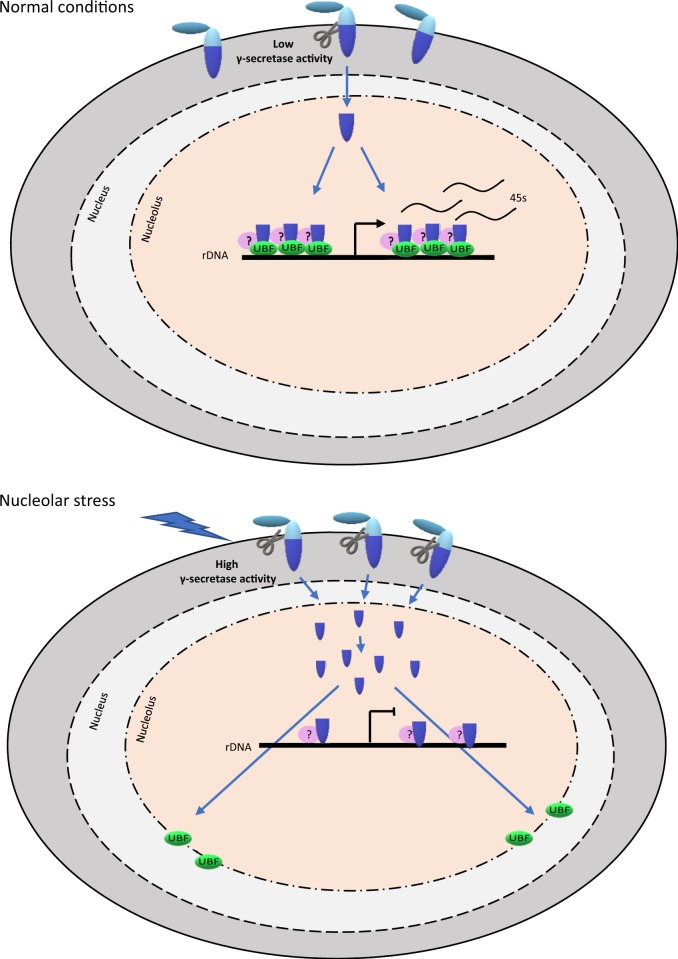
Schematic model showing the effect of β-DG ICD on rRNA synthesis in response to nucleolar stress. **a** Under normal conditions, β-DG ICD locates in nucleoli through its interaction with UBF, although additional proteins might be involved as well. **b** The nucleolar stress response triggers ɤ-secretase-mediated β-DG ICD cleavage and its further accumulation in the nucleolus. Increased β-DG ICD content inhibits rRNA synthesis by affecting both the localization and protein levels of UBF

The results raise the question of how β-DG ICD exerts a negative regulation on rDNA expression. β-DG lacks DNA-binding motifs or enzymatic activity and is considered as a scaffold protein, due to the presence of protein modules supporting interactions with diverse proteins (e.g. SH3, SH2, and WW domains)^5^. Therefore, we postulate that binding of β-DG ICD to UBF may lead to mislocalization and decreased levels of the latter protein, possibly by altering its stability, thereby interfering with the assembly of the Pol I initiation complex and the subsequent UBF-dependent activation of rDNA transcription (Fig. 9). Because the occupancy of rDNA regulatory regions by β-DG increased in response to nucleolar stress, it is also possible that β-DG ICD exerts its suppressive effect by displacing UBF from the Pol I transcription complex, which is consistent with mislocalization of UBF to the nucleolar periphery and cytoplasm that was observed in H_2_0_2_-treated cells. Further ChIP assays to ascertain whether β-DG and UBF coexist or not in the rDNA promoter region under nucleolar stress conditions are needed to solve this question. Furthermore, since β-DG has multiple partners in the nucleus, the existence of an indirect mechanism by which β-DG ICD represses UBF activity cannot be ruled out. The implication of β-DG ICD in rDNA expression could be envisioned as an extension of the homeostatic role of β-DG, where induction of ICD cleavage and its further targeting to the nucleolus in response to environmental stress, like oxidative stress, acts as a protective response to inhibit rDNA transcription and further proliferation of damaged cells.

In summary, our data are compatible with the paradigm that β-DG ICD functions as a negative regulator of rRNA transcription by impeding the transcriptional activity of UBF, as a part of the nucleolar stress response mechanisms that are activated under physiologically relevant stress conditions. Thus, in addition to its roles as PM and NE signaling scaffold, β-DG should be regarded as a regulator of Pol I transcription in the nucleolus.

## Materials and methods

### Cell culturing and treatments

Mouse C2C12 myoblasts (ATCC^®^ CRL-1772^™^) were grown as previously (Vásquez-Limeta, et al 2014). Generation of C2C12-derivative DG knockout cell lines with the CRISP/Cas9 system will be published elsewhere. For nucleoli disorganization treatments, cells were treated for 3 h with 0.01 μg/ml Act D (Sigma-Aldrich, St Louis, Missouri) or 25 µg/ml DRB (5,6 dichloro-1–d-ribofuranosylbenzimidazole; Sigma-Aldrich, St Louis, Missouri) or vehicle alone (DMSO), prior to confocal laser scanning microscopy (CLSM) analysis. The ɤ-secretase inhibitor N-(N-(3,5-difluorophenacetyl)-L-alanyl). S-phenylglycine t-butyl ester (DAPT; Sigma-Aldrich, St Louis, Missouri) was used at 10 µM for 24 h. For induction of the nucleolar stresses response, cells were cultured in DMEM supplemented with serum adjusted to pH 6, treated with 1.4 mM H_2_O_2_ (7722-84-1,Sigma-Aldrich) for 4 h or subjected to UV radiation using a pulse of 20 J/cm^2^ each 24 h for 2 days. For nuclease digestion assays, cells grown on coverslips were treated with either 100 μg/ml protease-free RNase A in CSK buffer (10 mM Pipes pH 6.8, 100 mM NaCl, 30 mM sucrose, 3 mM MgCl_2_, 1 mM EGTA, 0.5% (v/v) Triton X-100) for 40 min at 37 °C or with 200 μg/ml protease-free DNase I in PBS with 5 mM MgCl_2_ for 1 h at 37 °C, then the cells were fixed with PFA 4% and immunolabeled using primary antibodies and the corresponding fluorochrome*-*conjugated secondary antibodies. Negative control experiments were carried out by incubating cells only with CSK buffer (RNase) or PBS with 5 mM MgCl_2_ (DNase) prior to fixation.

### Plasmids and transfection

The expression vectors Flag-β-DG ICD WT, Flag-β-DG ICDY890E, and Flag-β-DG ICDY890A were generated by PCR amplification using DGWT-DG, DGY890E-GFP, and DGY890A-GFP plasmids as template, respectively^10^. The cDNA sequence encoding the intracellular domain of β-DG (ICD, amino acids 774–895) was amplified using an M-MLV reverse transcriptase coupled to a high-fidelity polymerase (*Pfu* turbo; Strategene) and primers containing Hind III and EcoRI restriction sites. Forward primer was 5′-CCTAAGCTTTATCGCAAGAAGAGGAAGGGC-3′ and the respective reverse primers were 5′-CTGAATTCTTAAGGGGGAACATACGGAGGG-3′ (ICD WT), 5′-CTGGAATCCTTAAGGGGGAAC**CTC**CGGAGGG-3′ (ICD Y890E mutation), and 5′-CTGAATTCTTAAGGGGGAAC**TGC**CGGAGGG-3′ (ICD Y890A mutation). PCR products were double digested with Hind III and EcoRI and cloned into the Hind III-EcoRI digested pFlag-CMV-10 vector. The integrity of the constructs was confirmed by DNA sequencing. Transfection was performed following supplier’s recommendations. Briefly, cells seeded onto glass coverslips were incubated overnight and then transfected with 3 μg of the corresponding vector premixed with 3 μl of Lipofectamine 2000 (Invitrogen, Carlsbad, CA, USA). Transiently transfected cells were analyzed at 24 h post-transfection. When indicated, cells were stably transfected by culturing them for 12 days in the presence of 2 µg/ml puromycin (Invitrogen) or for 5 days in the presence of 800 µg/ml gentamicin (G418), prior to being used for further experiments. For knockdown experiments, cells were stably transfected with psi-mH1 vector expressing a small interfering RNA (RNAi) specific for mouse DAG1 gene or a scrambled RNAi, as negative control (GeneCopoeia, Inc., Rockville, MD). For overexpression analysis, cells were stably transfected with GFP constructions as previously reported^9^.

### Antibodies

The following anti-β-DG primary antibodies were used: rabbit polyclonal antibodies G5 (Royuela et al, 2001) and Dystroglycan pTyr892 (Ilsley et al, 2002); goat polyclonal antibody C20 (Santa Cruz Biotechnology, CA), and mouse monoclonal antibodies MANDAG2^33^ and 7D11 (Santa Cruz). Rabbit polyclonal antibodies anti-B23 (C19-R), anti-Nup62 (H-122), anti-calnexin (H70), anti-fibrillarin (Ab5821), anti-GFP (sc8334) and mouse monoclonal antibodies anti-UBF (F9), anti-B23 (NPM1-FC-61991) (anti-RNA polymerase I (sc-46699), anti-lamin B1 (Ab16048), anti-and anti-RPA(sc-46699), and α-Tubulin (sc-32293) were purchased from Santa Cruz Biotechnology, CA, USA. Rabbit polyclonal anti-Flag (2368) and rabbit monoclonal anti-Notch1 (4147) antibodies were acquired from cell signaling, while mouse monoclonal anti-actin antibody was a gift from Dr. Manuel Hernández (CINVESTAV, Mexico City).

### Immunofluorescence and confocal microscopy analysis

Cells grown on coverslips were fixed with 4% paraformaldehyde for 10 min in PBS, permeabilized with 0.2% Triton X-100-PBS, blocked with 0.5% fetal bovine serum and 3% bovine serum albumin (BSA) in PBS and incubated overnight at 4 °C with the appropriate primary antibodies. The following day, cells were washed with 0.2% Triton X-100-PBS for 5 min and then with PBS alone three times, prior to be incubated for 1 h at room temperature with the appropriate fluorochrome*-*conjugated secondary antibody. For double immunolabeled samples, this was followed by overnight incubation at 4 °C with corresponding primary antibodies and the next day, cells were incubated with secondary fluorochrome*-*conjugated antibodies. Where indicated F-actin was labeled using TRITC-conjugated Phalloidin (Sigma-Aldrich St. Louis, Mo. USA) diluted 1:500 in PBS for 10 min at room temperature. For nuclease digestion assays, cells grown on coverslips were treated with either 100 μg/ml protease-free RNase A in CSK buffer (10 mM Pipes pH 6.8, 100 mM NaCl, 30 mM sucrose, 3 mM MgCl_2_, 1 mM EGTA, 0.5% (v/v) Triton X-100) for 40 min at 37 °C or with 200 μg/ml protease-free DNase I in PBS with 5 mM MgCl2 for 1 h at 37 °C. Cells were then immunolabeled using primary antibodies and the corresponding fluorochrome*-*conjugated secondary antibodies. Finally, coverslip preparations were incubated for 20 min at room temperature with 0.2 µg/ml diamidino-2-phenylindole (DAPI; Sigma-Aldrich) for nuclei visualization, mounted on microscope slides with VectaShield (Vector Laboratories Inc. Burlingame, CA, USA) and further analyzed by CLSM (TCP-SP5, Leica Microsystems, Heidelberg, Germany) using a Plan Neo Fluor 63 × (NA = 1.4) oil-immersion objective. Analyses of digitized images were carried out using ImageJ 1.62 software. The nucleolar area (μm^2^) of 600 nucleoli was analyzed in maxima projection using 3D objects counter and the data were analyzed using Prism6 software. Manders overlap coefficients were calculated for double labeling inmunofluorescences, using red (Alexa 594 nm) and green (FITC 488 nm) channels and the ImageJ plugin JACoB. A line intensity scan analysis was performed by ROI manager Multiplot.

### Western blotting

Cell lysates were electrophoresed on 10% SDS-polyacrylamide gels and transferred onto nitrocellulose membranes (Hybond-Nb, Amersham Pharmacia, GE Healthcare, Bukinghamshire, UK). Membranes were blocked in TBST (100 mM Tris-HCl pH 8.0, 150 mM NaCl, 0.5 % (v/v) Tween-20) with low fat-dried milk and then incubated overnight at 4 °C with the appropriate primary antibodies. The specific protein signal was developed using the corresponding secondary antibodies and enhanced chemiluminescence western blotting detection system (ECL^TM^; Amersham Pharmacia, GE Healthcare), according to the manufacturer's instructions.

### Cell fractionation

Total cell extracts were obtained with lysis Buffer (10 mM Tris-HCl pH 8, 30 mM NaCl, 0.2% Triton X-100, 4 mM Na_3_O_4_V, 50 mM NaF, 20 mM Na_2_MoO_4_, 1 mM PMSF, 1X complete protease inhibitor) sonicated three times at 3.5 μm with 15 s bursts and analyzed by western blotting. Purification of the cytosolic, nuclear, and nucleolar fractions was performed as previously reported^34^. A total amount of fifteen 100 mm dishes grown to 90% confluence were used; cells were washed twice with 1 ml of ice-cold PBS and collected by centrifugation at 15,000 rpm for 15 min at 4 °C. The pellet was resuspended in 1 ml of buffer TM (10 mM Tris-HCl pH 8, 2 mM MgCl_2_, 25 mM NaF, 10 mM Na_2_MoO_4_, 2 mM Na_3_VO_4_, 1 mM PMSF), supplemented with 1× complete protease inhibitor mixture (Roche Applied Science, Indianapolis, USA), and incubated on ice for 10 min. Then, 2% Triton X*-*100*-*PBS was added, and the homogenate incubated for 10 min on ice, transferred to a glass Dounce homogenizer, stroked 30 times with B pestle, and centrifuged at 5,000 rpm for 15 min at 4 °C. The supernatant was recovered as the cytosolic fraction and the nuclear pellet was resuspended in 1 ml of buffer I (0.32 M Sucrose, 3 mM CaCl_2_, 2 mM Mg(CH_3_COO)_2_, 0.1 mM EDTA, 10 mM Tris-HCl pH 8, 1 mM DTT, 0.5 mM PMSF, 0.5% (v/v) NP40) and 1 ml of Sucrose buffer II (2 M Sucrose, 5 mM Mg (CH_3_COO)_2_, 0.1 mM EDTA, 10 mM Tris-HCl pH 8.0, 1 mM DTT, 0.5 mM PMSF), and further purified by sucrose gradient centrifugation at 16,000 rpm for 1 h at 4 °C. The pellet was resuspended in 600 µl of buffer III (0.34 M Sucrose, 1 mM MgCl_2_, 0.1 mM EDTA, 10 mM Tris-HCl pH 8.0, 1 mM DTT, 0.5 mM PMSF) and aliquots collected for extraction of nuclear and nucleolar proteins. For nuclear proteins, the aliquot was centrifuged at 1,000 rpm for 5 min at 4 °C and the pellet resuspended in 200 µl of lysis buffer (50 mM Tris-HCl pH 8.0, 150 mM NaCl, 1 mM PMSF, 1% (v/v) Triton X-100), supplemented with protease inhibitor cocktail and phosphatase inhibitors, sonicated 3 times at 4μm with 15 s bursts and 4 min on ice between intervals, and then pre-cleared at 13,000 rpm for 2 min at 4 °C. For purification of nucleoli, the remaining 350 µl aliquot was sonicated 5 times at 5 μm with 30 s bursts and analyzed under a light microscope to ensure nucleoli integrity, prior to purification by centrifugation at 3,000 g for 20 min at 4 °C through a sucrose gradient, using buffer III and four volumes of Sucrose buffer IV (0.88 M Sucrose, 0.1 mM EDTA, 10 mM Tris-HCl pH 8.0, 1 mM DTT, 0.5 mM PMSF). Finally, the pellet was washed in 500 µl of buffer III and centrifuged at 2,000 g for 2 min at 4 °C, to save the supernatant as the nucleolar fraction^34^.

### Immunoprecipitation

Recombinant protein G-agarose beads (10 μl per sample; Invitrogen, Carlsbab, CA, USA) were equilibrated by gently agitation in lysis buffer (50 mM Tris-HCl pH 8, 150 mM NaCl, 1% Triton X-100, 2 mM Na_3_VO_4_, 25 mM NaF, 10 mM Na_2_MoO_4_, 1 mM PMSF, and 1× complete protease inhibitor mixture). We quantified 500 ug of protein and the lysates were pre-cleared with equilibrated beads for 2 h at 4 °C, the beads were removed by centrifugation at 3,500 rpm for 5 min and the pre-cleared extracts incubated overnight at 4 °C with the appropriate antibody. Incubations with an irrelevant IgG antibody were carried out in parallel. Thereafter, equilibrated protein G-agarose beads blocked previously with 4% BSA were added to the lysates and incubated overnight at 4 °C. Immune complexes were collected by centrifugation at 3,500 rpm for 5 min, washed twice for 10 min with 500 µl of washing buffer (50 mM Tris-HCl, 150 mM NaCl, 5 mM EDTA, 1% Tritón X-100, 1X complete protease inhibitor, 1 mM PMSF). Bound proteins were eluted from beads by boiling in sample buffer (50 mM Tris-HCl pH 6.8, 2% (w/v) SDS, 10% (v/v) glycerol, 0.1% (v/v) 2-mercaptoethanol, 0.001% bromophenol blue), prior to being subjected to western blot analysis. C2C12 cell lysates expressing GFP-tagged proteins were subjected to IP using the GFP-Trap® system (Chromotek, Germany), according to the manufacturer’s instructions.

### Quantitative reverse PCR (RT-qPCR)

For analysis of rRNA transcripts, total RNA was extracted using Direct-zol ^TM^ RNA Miniprep Kit (Zymo Research, Irvine, CA, USA), according to the manufacturer's instructions. And further analyzed by qRT-PCR with specific primers amplifying 45 S pre-rRNA (forward, 5′-GTGTCCAAGTGTTCATGCCA; reverse, 5′-CGATCTAAGAGTGAGCAACGAC), 18 S rRNA (forward, 5′-TTCCGACCATAAACGATGCC; reverse, 5′-GCTCCACCAACTAAGAACGG), 28 S rRNA (forward, 5′-GATGGTGAACTATGCTTGGG; reverse, 5′-GAATAGGTTGAGATCGTTTCGG), and GAPDH mRNA (forward, 5′-CTTGGGCTACACTGAGGACC; reverse, 5′-CTGTTGCTGTAGCCGTATTC). For analysis of *DAG1* mRNA expression, the following primers were used: forward, 5′-GAGATCATCAAGGTGTCTGCA and reverse, 5′-GTGGCTCATTGTGGTCTTCAG. RT-qPCR reactions were carried out in the StepOneplus System (Life technologies), using KAPA SYBR® FAST One-Step qRT-PCR Master Mix (2 × ) Kit, and further analyzed with the StepOne Software v2.3. The data were analyzed by comparative method 2^−ΔΔ*CT*^.

### RNA pol I reporter assays

C2C12 cells stably expressing GFP-ICD-βDG or GFP alone were co-transfected with both the Renilla Luciferase vector (transcribed by RNA polymerase II) as a control to normalize for differences in transfection efficiency and with the firefly luciferase RNA polymerase I reporter vector (pMrTsp-9-T10). The two vectors were kindly donated by Gernot Längst Lab., Regensburg University, Germany. After 48 h, the luciferase activity was measured using Dual Luciferase Assay kit (Promega). The firefly luciferase counts of the RNA polymerase I reporter were divided by the Renilla luciferase counts and compared to GFP control transfections.

### Cell proliferation assays

C2C12 cells stably expressing GFP-ICD-βDG or GFP alone were harvested and plated in triplicate onto 12 wells microplates at 1 × 10^3^ cells/mL confluence. Proliferation was measured for 13 days using the MTT (3-(4,5-dimethylthiazole)− 2–5-diphenyl tetrazolium bromide) commercial kit (Sigma-Aldrich), following the manufacturer’s instructions.

### Chromatin immunoprecipitation assays

C2C12 cells were incubated with 1% formaldehyde in PBS for 10 min at room temperature. Cross-link reaction was stopped by adding glycine to a final concentration of 125 mM. Cells were washed and harvested with cold PBS. After centrifugation at 1500 g, the pellet was resuspended in sonication buffer (50 mM Hepes pH 7.9, 140 mM NaCl, 1 mM EDTA, 1% Triton X-100, 0.1% sodium deoxycholate, 0.1% SDS and protease inhibitors). Soluble and sheared chromatin were obtained by applying five cycles of sonication on ice at 10% of amplitude for 20 s (Q55 sonicator, Qsonica) and recovered by centrifugation. Soluble chromatin was pre-clarified by mixing with 50 µl of recombinant protein G-agarose beads (rPGA Invitrogen) and incubated with shaking for 1 h at 4 °C. The mix was centrifuged at 3,000 × g for 1 min to remove rPGA. Twenty five micrograms of pre-clarified chromatin diluted 1:10 in sonication buffer were mixed with 7.8 µg of mouse purified serum anti-β-dystroglycan or 7.8 µg of mouse IgG (irrelevant control). Finally 25 µl of rPGA were added to each sample and incubate with shaking overnight at 4 °C. Immunoprecipitated DNA-protein complexes were extensively and sequentially washed with sonication buffer, wash buffer A (50 mM Hepes pH 7.9, 500 mM NaCl, 1 mM EDTA, 1% Triton X-100, 0.1% sodium deoxycholate, 0.1% SDS), wash buffer B (20 mM Tris-Cl pH 8, 1 mM EDTA, 250 mM LiCl, 0.5% NP40, 0.5% deoxycholate), and TE buffer. Finally, the pellet was incubated with shaking for 10 min at 65 °C in elution buffer (50 mM Tris-Cl pH 8, 1 mM EDTA, 1% SDS, 50 mM NaHCO_3_). Supernatant was recovered by centrifugation and the cross-link reversion was carried out by adding RNAase A and Proteinase K to each sample and incubating with shaking for 4 h at 65 °C. DNA was recovered by phenol–chloroform extraction and ethanol precipitation. Twenty nanograms of DNA was used to perform quantitative PCR (qPCR), using SYBR green and rDNA-specific primers as previously reported^35,36^.

### Ribosomal profile analysis

Cells grown at 80–90% confluency were harvested, washed with PBS with 100 μg/mL cycloheximide and centrifuged at 5,000 rpm for 5 min at 4 °C, prior to suspension in 200 μl of lysis buffer containing 100 μg/mL cycloheximide and 1× complete protease inhibitor mixture. Thereafter cells were centrifuged for 10 min at 10,000 rpm and the supernatant was layered on top of a 15–50% sucrose gradient to be centrifuged at 25,000 rpm for 5:30 h at 4 °C in a Beckman SW28Ti rotor. All gradients were scanned at 260 nm from the top with an ISCO gradient collector. Collected fractions were precipitated with ethanol and centrifuged at 15,000 rpm for 30 min to obtain ribosomal particles.

## Supplementary information

Supplemental Figures

Supplemental figure legends
