# Supplementary figures and images for: The intracellular domain of β-dystroglycan mediates the nucleolar stress response by suppressing UBF transcriptional activity
Source: Cell Death Dis. 2019 Feb 27;10(3):196. doi: 10.1038/s41419-019-1454-z (PMC6393529; doi:10.1038/s41419-019-1454-z)

Supplementary Figure 1

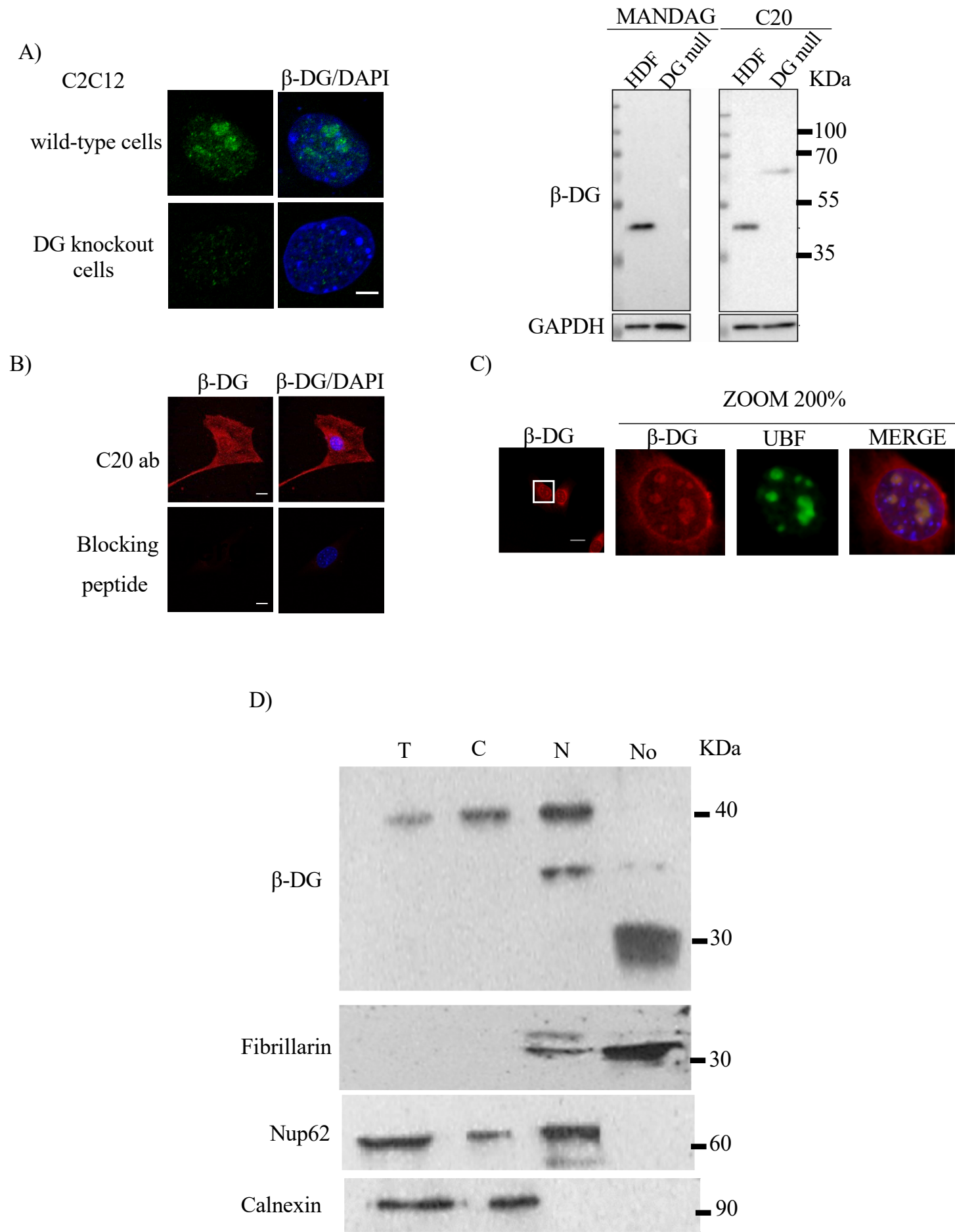

Supplementary Figure 2.

A)

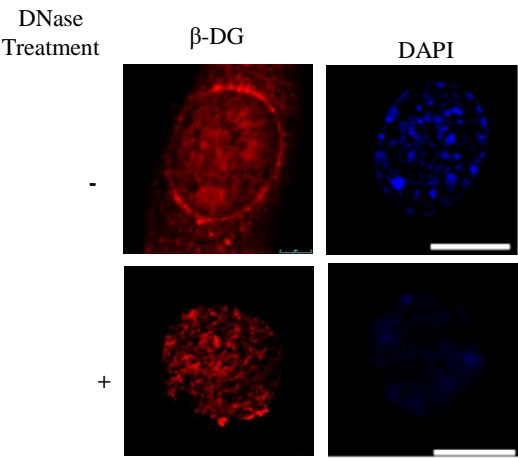

B)

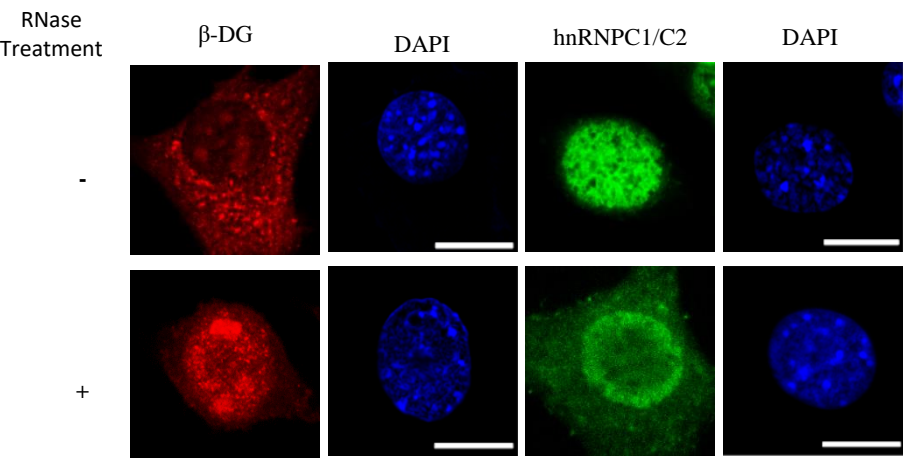

Supplementary Figure 3.

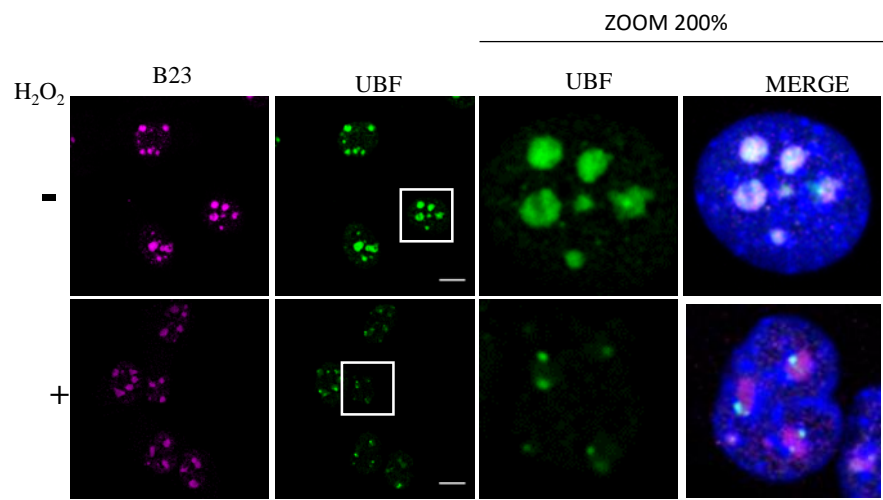

Supplementary Figure 4

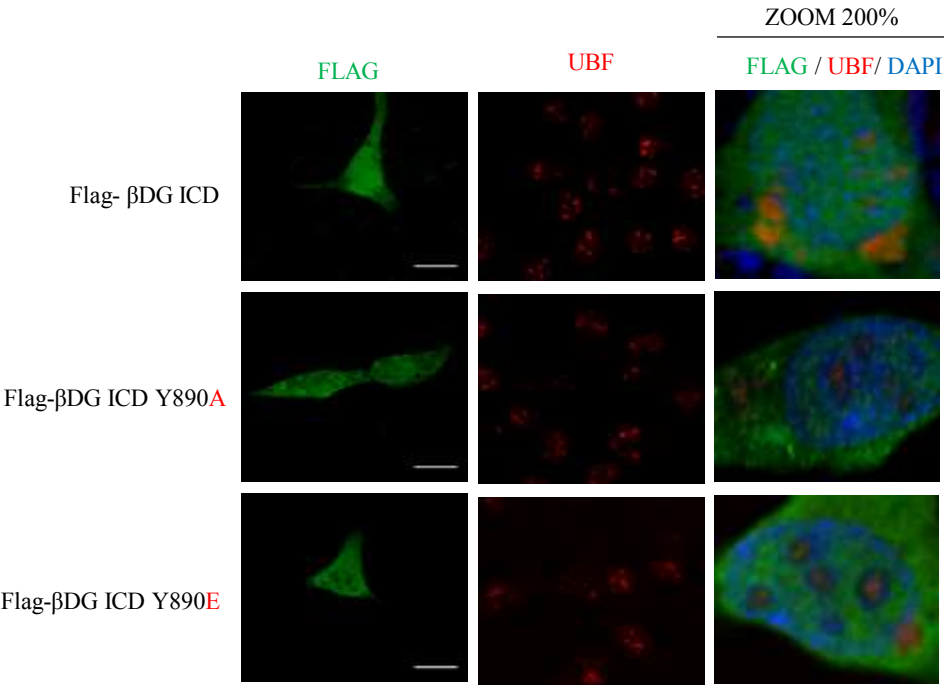

Supplement: Supplementary file 1 — Supplemental Figures [file 41419_2019_1454_MOESM1_ESM.pdf]
