# Supplementary material for: The intracellular domain of β-dystroglycan mediates the nucleolar stress response by suppressing UBF transcriptional activity
Source: Cell Death Dis. 2019 Feb 27;10(3):196. doi: 10.1038/s41419-019-1454-z (PMC6393529; doi:10.1038/s41419-019-1454-z)
Supplement: Supplementary file 2 — Supplemental figure legends [file 41419_2019_1454_MOESM2_ESM.docx]

**Supplementary Figure 1. β-DG is present in the nucleolus**. (A) To demonstrate C20 antibody specificity, wild type and DG knockout C2C12 cells were subjected to immunofluorescence assays (left panel). Human dermal fibroblast from a healthy subject (HDF) and from a patient with Walker-Warburg syndrome (DG null) were subjected to SDS-PAGE/WB analysis, using the anti-β-DG antibodies C20 and MANDAG. GAPDH was used as loading control (right panel). (B) C2C12 wild type cells were pre-incubated with C20 blocking peptide prior to be subjected immunofluorescence assays. (A and B) Nuclei were labeled with DAPI prior to be subjected to CLSM analysis. (C) An additional anti-β-DG antibody (G5 rabbit polyclonal) was used to confirm nucleolar localization of β-DG. C2C12 cells were subjected to double immunofluorescence assays to show colocalization of β-DG and the nucleolar protein UBF. Nuclei were stained with DAPI before CLSM analysis. Representative single Z-sections are shown (scale bar = 10 µm). (D) The presence of the β-DG proteolytic fragment (~30 kDa) in the nucleolar fraction of C2C12 cells was confirmed using an alternative β-DG antibody (C20, goat-polyclonal antibody). Total (T), cytoplasm (C), nuclear (N) and nucleolar fractions (No) from C2C12 cells were subjected to SDS-PAGE/ WB analysis using primary antibodies against β-DG. Fibrillarin, Nup 62 and calnexin served as purity markers for nucleolus, nucleus and cytoplasm respectively.

**Supplementary Figure 2.** **β-DG Nucleolar localization depends on DNA.** C2C12 myoblasts grown on coverslips were treated with 100 μg/ml protease-free RNase A in CSK buffer for 40 min at 37ºC or with 200 μg/ml protease-free DNase I in PBS with 5 mM MgCl_2_ for 1 h at 37ºC prior to fixation with PFA 4%. Treated cells were immunostained for β-DG and counterstained with DAPI to visualize DNA or immunolabeling for hnRNP C1/C2 (RNA-binding protein) to track RNA degradation. (-) control experiments were carried out by incubating cells only with CSK buffer (RNase) or PBS with 5 mM MgCl_2_ (DNase) prior to fixation. Immunofluorescence was analyzed by CLSM. Scale bar, 5 μm.

**Supplementary Figure 3. Effect of H_2_O_2_ treatment on the distribution of nucleolar proteins.** C2C12 cells were treated with H_2_O_2_, prior to being subjected to double immunostaining with antibodies against B23 and UBF. Cells were labeled then with DAPI to visualize nuclei prior to be analyzed by CLSM. Scale bar, 10 µM

**Supplementary Figure 4. Overexpression of ICD β-DG affects UBF independently of its phosphorylation status.** C2C12 cells were transiently transfected to express FLAG-β-DG ICD or its mutant variants FLAG-β-DG ICD Y890A and FLAG-β-DG ICD Y890E. At 24 post-transfection cells were double immunostained using antibodies against FLAG-epitope and UBF. Upon labeling of nuclei with DAPI, the cell preparations were analyzed by CLSM. Scale bar, 10 µM.
